# A Systematic Review and Meta-Analysis of Open vs. Laparoscopic Resection of Gastric Gastrointestinal Stromal Tumors

**DOI:** 10.14740/jocmr1547w

**Published:** 2015-03-01

**Authors:** Jean-Sebastien Pelletier, Richdeep S. Gill, Sayf Gazala, Shahzeer Karmali

**Affiliations:** a Department of Surgery, University of Alberta, Edmonton, Alberta, Canada; b Center for the Advancement of Minimally Invasive Surgery (CAMIS), Royal Alexandria Hospital, Edmonton, Alberta, Canada

**Keywords:** GIST, Laparoscopy, Resection, Stomach, Surgery, Review, Meta-analysis

## Abstract

Gastric gastrointestinal stromal tumors (GISTs) are the most common sarcoma of the gastrointestinal tract, and surgical resection is the primary treatment of early disease. Limited data exist concerning laparoscopic resections of these neoplasms. This systematic review was designed to evaluate the literature comparing laparoscopic and open surgical resection of gastric GISTs and to assess the effectiveness and safety of this minimally invasive technique. We performed a systematic search of MEDLINE, the Cochrane Library, PubMed, Embase, Scopus, Web of Science, Google Scholar, the clinical trials database and ProQuest Dissertations and Theses as well as the past 3 years of conference abstracts from the Society of American Gastrointestinal and Endoscopic Surgeons Annual Meetings. Studies comparing the open and the laparoscopic approaches to the resection of gastric GISTs were included in this systematic review. Two reviewers independently performed the screen of titles and abstracts, the full manuscript review, the data extraction and the risk of bias assessment. A quantitative analysis was performed. Of the 189 studies identified, seven studies were included. The laparoscopic approach was associated with a significantly lower length of hospital stay (3.82 days (2.14 - 5.49)). There was no observed difference in operative time, adverse events, estimated blood loss, overall survival and recurrence rates. This study supports that laparoscopic resection is safe and effective for gastric GISTs and is associated with a significantly lower length of hospital stay. Further trials are needed for cost analysis and to rigorously assess oncologic outcomes.

## Introduction

Gastrointestinal stromal tumors (GISTs) are the most common gastrointestinal sarcoma. Initially believed to arise from smooth muscle cells, it is now known that GISTs are malignancies that arise from the interstitial cells of Cajal, which are pacemaker cells found in the bowel wall [1]. These tumors can be found anywhere within the gastrointestinal tract, but the stomach is its most common location, accounting for at least half of them [2].

Substantial advances have been made in the medical treatment of these tumors as tyrosine kinase inhibitors have enjoyed great success in their treatment. It in fact stands as the foremost example of the therapeutic potential of targeted therapy aimed at receptor inhibition in medical oncology [2]. That being said, surgical resection is still the most important component in the treatment of resectable, non-metastatic GISTs [3]. Also, with the aforementioned advances made in the medical treatment of these tumors, there is the possibility of a future broadening of surgical indications to include patients with metastatic disease, but this currently remains investigational [4].

A growing trend in gastrointestinal surgery has been the widespread adoption of minimally invasive procedures. It has been shown to be equally efficacious for a variety of surgical conditions, and confers advantages in terms of reduced pain and shorter length of hospital stay, to name a few [5]. Additionally, for certain cancers, it has been shown to be just as effective as the open technique to achieve excellent oncologic results [6]. The surgical resection of GISTs seems to lend itself very well to laparoscopic resections, as unlike other gastric malignancies, wide margins and lymph node dissections are not necessary in their surgical management [1]. While these factors facilitate their laparoscopic resection, great care must be taken when handling these tumors as rupture of their capsule confers a near 100% risk of recurrence [7]. To date, only a few small studies have compared laparoscopic to open resections for GISTs and the efficacy and safety of laparoscopic resections has not been confirmed. Therefore, we aimed to systematically review the current literature comparing laparoscopic to open surgical resection of GISTs of the stomach.

### Objectives

The objectives of this systematic review are to evaluate the literature comparing laparoscopic and open surgical resection of gastric GISTs to assess the effectiveness and safety of this minimally invasive technique.

## Materials and Methods

A protocol was developed for this systematic review prior to the collection and analysis of data. This protocol was registered through PROSPERO, the international prospective register of systematic reviews. The registration number for this study’s protocol is CRD42012002163, and can be accessed electronically at: http://www.crd.york.ac.uk/prospero.

The eligibility criterion for this review included all English language studies involving adult patients undergoing surgical resection of GISTs of the stomach. The interventions of interest were open and laparoscopic surgical resections of gastric GISTs.

The primary outcome measures included length of hospital stay, operative time and adverse events. Secondary outcomes included intra-operative blood loss, pain scores, conversion rate and oncologic outcomes, namely overall survival, disease-specific survival and recurrence.

Any study design that included a comparison of the two operative approaches of interest was included. This included, but was not limited to randomized control trials, prospective and retrospective cohort studies and case-control studies. Case studies and case series were not included. We did not limit our search by time period and all searches are current up to February 7, 2012.

In order to perform this search, the following four search concepts were used: “laparoscopy”, “open resection”, “stomach” and “gastrointestinal stromal tumor”. The information sources that were searched included MEDLINE, the Cochrane Library, PubMed, Embase, Scopus, Web of Science and Google Scholar. Citations and references of the included studies were also studied. In an effort to include grey literature into our search, we performed a thorough search of conference abstracts from the Society of American Gastrointestinal and Endoscopic Surgeons Annual Meetings for the previous 3 years, the foremost organization examining the benefits of the laparoscopic surgical approach. Additionally, the clinical trials database and ProQuest Dissertations and Theses were searched for all relevant material. The electronic search strategy for the MEDLINE database is included in Table 1.

**Table 1:** Medline Search Strategy

Concept 1		Concept 2		Concept 3		Concept 4	Results
laparoscop* or exp laparoscopy or telesurg* or “tele surg*” or minimal* adj3 (invasiv* or access*) or celiosc*	AND	(usual or wedge or traditional or standard* or open or invasive or normal or routine) adj3 (resect* or surger* or surgi*)	AND	GIST or exp gastrointestinal stromal tumors/or (gastrointestinal stromal adj2 (neoplasm* or tumo*)).mp.	AND	gastri* or stomach	81

Limit to English language.

Once this search was completed, the study selection proceeded as follows. Following the removal of all duplicates, two reviewers (JSP and SG) independently performed the initial screen of titles and abstracts. If one of the two reviewers deemed the study acceptable, it proceeded to a full manuscript review. At this point, both primary reviewers (JSP and SG) independently performed a full manuscript review using pre-specified criteria. A third reviewer (RSG) resolved all of the discrepancies at this point, and had the final say as to which study was effectively included in our systematic review.

Both primary reviewers (JSP and SG) were then responsible for the data collection, which was done independently, using a standardized form. This form included all of the variables of interest, namely study characteristics, patient demographic data, tumor-specific factors, surgical factors as well as our outcome measures.

Additionally, all of our included studies underwent a risk of bias assessment, which was done independently by the two primary reviewers (JSP and SG). The Cochrane Collaboration’s tool for assessing risk of bias was to be used for randomized and quasi-randomized trials and the Newcastle-Ottawa scale for cohort studies and case-control trials. Once again, any disagreements were addressed by third party adjudication by a third reviewer (RSG).

The summary measures used were risk ratio for categorical data and difference in means for continuous data. When unavailable, mean values were computed from median values using the formulas outlined by Hozo et al [8]. When standard deviations were unavailable, they were computed using confidence intervals and P-values or imputed from ranges and inter-quartile ranges [9]. A random effects model was chosen in order to be more conservative in our estimates, and statistical heterogeneity was assessed using the I^2^ test statistic. Low statistical heterogeneity was defined as an I^2^ of less than 25%; moderate statistical heterogeneity was defined as an I^2^ of 50%; and high statistical heterogeneity was defined as an I^2^ of greater than 75%. Also, in order to assess for risk of publication bias, we chose to perform a visual funnel plot assessment, but only if there were at least 10 studies included in this systematic review.

In terms of additional analyses, we chose to perform a sensitivity analysis based on stage of disease *a priori*. Also, we chose to perform a sensitivity analysis comparing published abstracts to full studies, which was also decided prior to data collection. Review Manager software was used to perform the data analysis and to create the forest plot.

## Results

**Figure 1:**
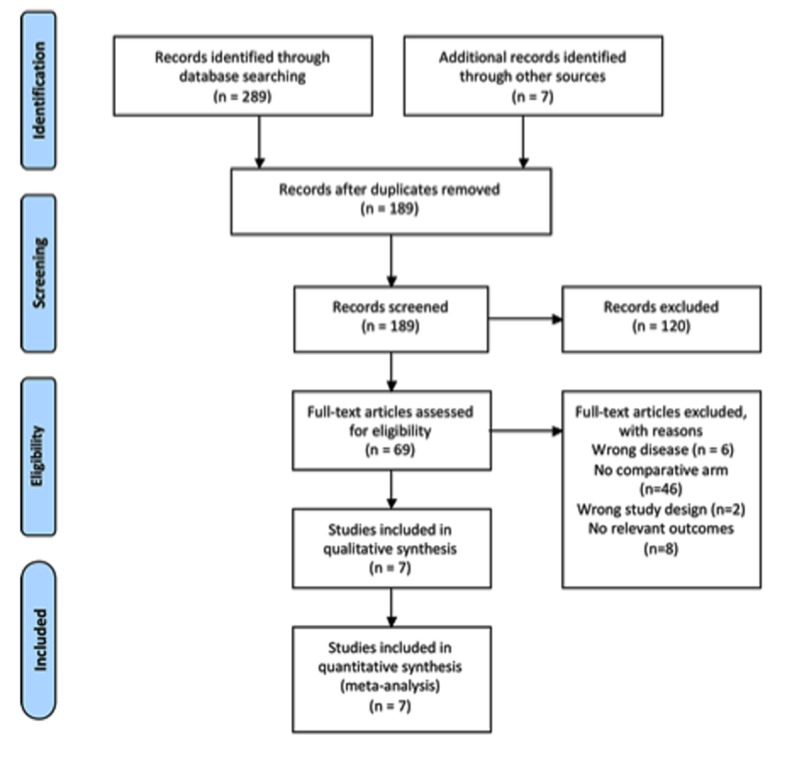
Systematic review PRISMA flow diagram.

A systematic review of the literature was completed using the four search concepts noted above. Our simplified flow diagram is included in Figure 1. The initial search of our databases yielded a total of 289 studies. A hand-search of the Society of American Gastrointestinal and Endoscopic Surgeons Annual Meetings for the three antecedent years added another seven studies. Duplicate studies were removed after which 189 studies remained.

Our initial screen of titles and abstracts excluded all studies that obviously did not evaluate laparoscopic surgery or gastric GISTs. Studies were also removed if they were clearly review articles. Sixty-nine studies then remained for full manuscript review. As a result of the manuscript review, seven studies with a total of 267 participants remained to be included in the quantitative and qualitative analysis [1, 3, 10-14]. The other studies were excluded because they lacked a comparative open surgery arm (n = 46), they did not present relevant outcomes (n = 8), they had an inappropriate study design (n = 2) or they studied the wrong disease process (n = 6). The study characteristics for our included studies are included in Table 2 [1, 3, 10-14] and the patient characteristics are included in Table 3.

**Table 2:** Study Characteristics

Authors	Year	Journal name	Article name	Design	Outcomes	Follow-up period (months)	
						L	O
Catena et al	2008 [1]	Journal of Gastrointestinal Surgery	Laparoscopic treatment of gastric GIST: report of 21 cases and literature’s review	RCS	LOS OR time Adverse events Conversion rate Overall survival EBL Recurrence rate	Mean: 35	Mean: 91
Nishimura et al	2007 [3]	Surgical Endoscopy	Surgical strategy for gastric gastrointestinal stromal tumors: laparoscopic vs. open resection	RCS	OR time Conversion rate EBL Recurrence rate	Median: 19	Median: 31
Karakousis et al	2011 [10]	Annals of Surgical Oncology	Laparoscopic versus open gastric resections for primary gastrointestinal stromal tumors (GISTs): a size-matched comparison	CCS	LOS OR time Adverse events Overall survival EBL Recurrence rate	Median: 28	Median: 43
Matthews et al	2002 [11]	Surgical Endoscopy	Laparoscopic vs. open resection of gastric stromal tumors	RCS	LOS OR time Adverse events EBL Recurrence rate	Mean: 20	Mean: 18
Pitsinis et al	2007 [12]	Hepato-Gastroenterology	Single center experience of laparoscopic vs. open resection for gastrointestinal stromal tumors of the stomach	RCS	LOS OR time Adverse events Recurrence rate	Median: 9	Median: 9
Silberhumer et al	2009 [13]	Journal of Gastrointestinal Surgery	Surgery for gastrointestinal stromal tumors of the stomach	RCS	LOS Adverse events Conversion rate Recurrence rate	Mean: 30	Mean: 41
Wu et al	2010 [14]	Journal of Laparoscopic and Advanced Surgical Techniques	Gasless laparoscopy-assisted versus open resection for gastrointestinal stromal tumors of the upper stomach: preliminary results	RCS	LOS OR time Adverse events Pain score Conversion rate Overall survival EBL	N/A	N/A

RCS: retrospective cohort study; CCS: case-control study; BMI: body mass index; LOS: length of stay; OR time: operating room time; EBL: estimated blood loss; Med: median; N/A: not available; L: laparoscopic group; O: open group.

**Table 3:** Patient Characteristics of Included Trials

Authors, year	Number of patients		Age		BMI		Tumor size		Procedures performed	
	L	O	L	O	L	O	L	O	L	O
Catena et al, 2008 [1]	21	25	50.1	54.6	N/A	N/A	4.5	6.2	86% W; 14% DG	50% W; 33% DG; 17% PG
Nishimura et al, 2007 [3]	39	28	62^t^	63^t^	N/A	N/A	3.8^t^	4.2^t^	100% W	68% W; 4% DG; 18% PG; 11% TG
Karakousis et al, 2011 [10]	40	40	67^t^	70^t^	N/A	N/A	3.6^t^	4.3^t^	100% W	95% W; 5% DG
Matthews et al, 2002 [11]	21	12	53.9	50.5	N/A	N/A	4.5	4.9	86% W; 14% DG	50% W; 33% DG; 17% PG
Pitsinis et al, 2007 [12]	6	7	70^t^	68^t^	N/A	N/A	5^t^	11.5^t^	N/A	N/A
Silberhumer et al, 2009 [13]	22	41	61.3	62.5	N/A	N/A	3.5	5.8	100% W	78% W; 12% DG; 10% TG
Wu et al, 2010[14]	15	13	61.6	60.7	23.4	22.7	2.6	2.5	100% W	100% W

Values expressed as mean unless otherwise indicated. ^t^: median value. L: laparoscopic group; O: open group; N/A: not available; W: wedge resection; DG: distal gastrectomy; PG: proximal gastrectomy; TG: total gastrectomy.

An assessment of risk of bias within studies was performed, but due to the fact that we only captured observational studies, the Newcastle-Ottawa scale was used for all of our studies. Six of our seven studies had a score of seven out of nine, with the other getting a score of 8. For all of our studies, the points were lost on the comparability analysis, due to the fact that their study design did not allow them to control for important factors. For the study by Karakousis et al [10], they did control for tumor size, which we considered to be an important factor. The result of our risk of bias assessment is shown in Table 4.

**Table 4:** Newcastle-Ottawa Scale for Risk of Bias

Study ID	Catena et al, 2008 [1]	Nishimura et al, 2007 [3]	Karakousis et al, 2011 [10]	Matthews et al, 2002 [11]	Pitisinis et al, 2007 [12]	Silberhumer et al, 2009 [13]	Wu et al, 2012 [14]
Study design	Retrospective cohort	Retrospective cohort	Case-control study	Retrospective cohort	Retrospective cohort	Retrospective cohort	Retrospective cohort
Selection	****	****	****	****	****	****	****
Comparability			*				
Outcome/exposure	***	***	***	***	***	***	***

**Figure 2:**
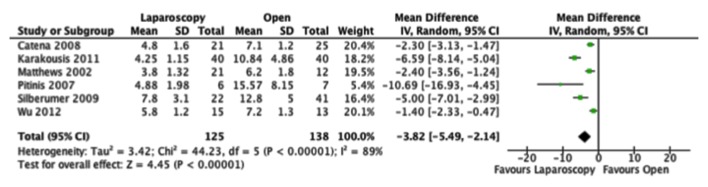
Forest plot depicting the mean differences in length of hospital stay (days) in the included studies, if reported.

For our primary outcome measures, we were able to include six studies into the analysis. All of the studies that reported on length of hospital stay found that there was at least a trend towards improvement in the laparoscopic group. When the data were combined, we found that the laparoscopic group stayed in hospital on average 3.82 days less, which is both clinically and statistically significant. There was high statistical heterogeneity however, with an I^2^ of 89% (Fig. 2).

**Figure 3:**
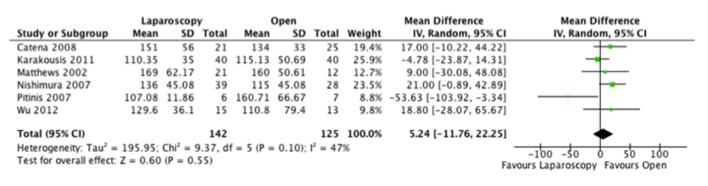
Forest plot depicting the mean differences in length of operative time (min) in the included studies, if reported.

As for operative time, the results were varied with two studies favoring the laparoscopic and four the open approach. When we combined the results, the resultant mean difference between both approaches was not clinically or statistically significant (Fig. 3).

**Figure 4:**
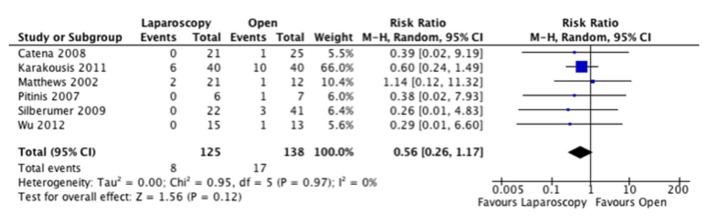
Forest plot depicting the risk ratio of total adverse events in the included studies, if reported.

Adverse events were inconsistently reported in these studies and the classification of these events was poorly defined. As such, we were only able to compare total adverse events that were noted in these studies. Five of six studies had lower adverse event rates recorded in the laparoscopic group, but when the information was combined, we did not find a significant difference between the approaches (Fig. 4).

**Figure 5:**
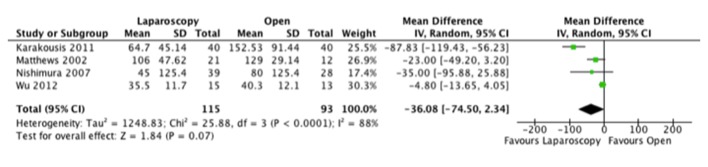
Forest plot depicting the mean differences in the estimated blood loss (mL) in the included studies, if reported.

As for our secondary outcomes, four studies [1, 3, 13, 14] reported on their conversion rate from laparoscopic to open, with five conversions out of 97 operations for a conversion rate of 5.2%. A pain assessment was only performed in one of the included studies [14], and they did find that patients undergoing laparoscopic surgery had significantly less pain in the first 3 days post-operatively. Pain was then found to be the equivalent in both groups on the forth post-operative day. Four studies reported on estimated blood loss, and no difference was found between the open and the laparoscopic group (Fig. 5).

**Figure 6:**
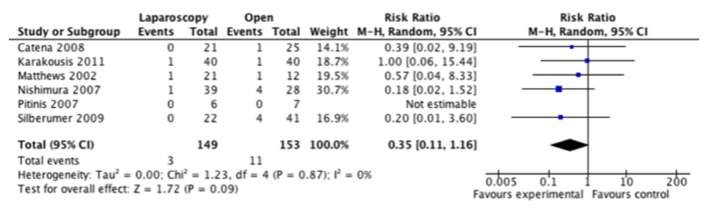
Forest plot depicting the risk ratio for recurrence in the included studies, if reported.

**Figure 7:**
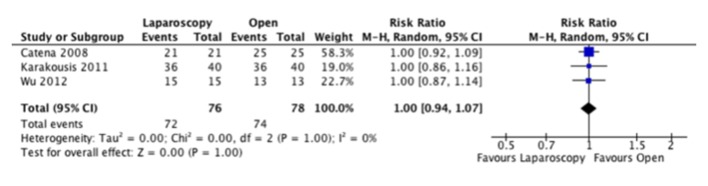
Forest plot depicting the risk ratio for overall survival in the included studies, if reported.

Oncologic outcomes were inconsistently reported in our included studies. Additionally, the follow-up was not consistent across both groups with a mean follow-up of 26.5 months and of 43.6 months for the laparoscopic and open groups respectively. Recurrence rates were found to be equal between groups in one study, and lower in the laparoscopic group for five studies. When combined however, this was not found to be significant (Fig. 6). Disease-specific survival was not assessed due to a lack of data. We were able to assess overall survival from three studies, which did not show a difference between groups (Fig. 7).

We did not perform a funnel plot for risk of bias, as there were less than 10 studies included in our review. Also, stage of disease was not reported in any of the included studies, and we were therefore unable to complete our aforementioned sub-group analysis. Consideration was given to completing a subgroup analysis based on tumor size, which was reported in all studies as well as tumor risk, as this was reported in four studies. However, as the results were not reported taking these variables into consideration, these were not possible. Similarly, we were unable to perform a sensitivity analysis comparing published abstracts and studies, as none of our included studies were published abstracts.

## Discussion

The laparoscopic approach to surgical conditions has been of significant benefit and is one of the most important advances in surgical technology in recent history. This systematic review and meta-analysis adds to the wealth of evidence supporting these techniques as it demonstrated a statistically significant and clinically relevant benefit in terms of length of hospital stay. Specifically, patients undergoing open surgical resection were in hospital for 3.8 days longer than those who underwent a laparoscopic procedure. This is a significant finding, as it not only improves patient satisfaction, but it also implies improved patient wellness, as they are willing to return to their home environment sooner. Additionally, while it is known that laparoscopic surgery is associated with a higher intra-operative cost [15], previous studies have shown that this is compensated by other benefits, including a shorter length of stay, which makes minimally invasive approaches cost equivalent [16] or economically beneficial [17] as compared to traditional open approaches. A cost benefit analysis would have to be performed for gastric GISTs to accurately assess this.

Our review also suggested that laparoscopic resections for gastric GISTs are feasible with a conversion rate of 5%, which is similar to other studies on laparoscopic gastric surgery [18-20]. Also, the laparoscopic approach was shown to be safe, with no difference in adverse events or intra-operative blood loss. Additionally, the one included study that reported on post-operative pain did report a significant improvement with the laparoscopic approach for the first 3 post-operative days [14].

Due to the paucity of data, it is difficult to come to a conclusion regarding oncologic outcomes. While recurrence rates trended towards being less in the laparoscopic group, this is likely influenced by the fact that the overall follow-up was longer in the open group, thus allowing for a longer amount of time to find recurrences. To adequately determine differences in recurrence rates, further higher quality prospective studies are needed.

Although none of the studies reported a significant difference in terms of tumor characteristics such as tumor size and extent of surgery, most of the included trials reported smaller tumors and less extensive surgery in the laparoscopic groups. Additionally, as the results were not presented with these variables taken into consideration, we were unable to perform a sensitivity analysis on these variables. The ability to perform laparoscopic resections of large GISTs requiring larger resections would have to be analyzed in the future.

The main limitation of this systematic review is the overall low quality of evidence of the included studies. We did not find any prospective randomized clinical trials that specifically addressed our question, and all of our included studies were retrospective analyses. Also, the included studies had a limited number of patients, with a total of only 267 patients across seven studies.

### Conclusions

Our meta-analysis demonstrates that laparoscopic surgery is safe in the surgical management of gastric GISTs, with the additional benefit of shorter length of hospital stay. Further prospective research is needed to clarify whether the laparoscopic approach is oncologically equivalent to the open approach.
